# Non-contrast 3D radial and QISS MRA for transcatheter aortic valve replacement planning

**DOI:** 10.1186/1532-429X-17-S1-O71

**Published:** 2015-02-03

**Authors:** Akos Varga-Szemes, Paola M Cannao, Giuseppe Muscogiuri, Matthias Renker, Carlo N De Cecco, Shivraman Giri, Davide Piccini, Daniel H Steinberg, Joseph U Schoepf

**Affiliations:** 1 Medical University of South Carolina, Charleston, SC, USA; 2 University of Milan, Milan, Italy; 3 University of Rome "Sapienza", Rome, Italy; 4 Siemens Healthcare IM BM IP, Lausanne, Switzerland; 5 University of Lausanne, Lausanne, Switzerland; 6 Siemens Medical Solutions, Chicago, IL, USA; 7 University Hospital Giessen and Marburg, Giessen, Germany

## Background

Because of the high prevalence of renal insufficiency in patients eligible for transcatheter aortic valve replacement (TAVR), a non-contrast evaluation of the aortic root complex along with the entire vascular access route is desirable for pre-procedural evaluation. In this pilot study we proposed to test two novel investigational, non-contrast MRA techniques to develop a protocol for TAVR planning.

## Methods

The study protocol was approved by the Institutional Review Board. Non-contrast MRA was performed in 7 subjects (5 healthy volunteers and 2 patients) on a 1.5T system (MAGNETOM Avanto, Siemens AG, Erlangen, Germany). A prototype 3D self-navigated whole-heart radial MRA acquisition based on a spiral phyllotaxis pattern was used to assess the cardiac anatomy and the aortic root (FOV 220mm^3^, TR/TE 3.1/1.5ms, flip angle 90°). This pulse sequence employs a superior-inferior bSSFP readout at the beginning of each heartbeat to correct the displacement in the k-space based on the blood pool signal. For the evaluation of the abdominal aorta and the femoral access route, both the 3D whole-heart (FOV 400mm^3^, TR/TE 3.1/1.5ms, flip angle 90°) and the prototype quiescent-interval single-shot (QISS) MRA pulse sequence were used (FOV 400x260mm^2^, TR/TE 3.5/1.4ms, flip angle 90°, acquisition length 144mm, number of stations 3-4). Aortic root, abdominal aorta and femoral runoff measurements were obtained and image quality was evaluated. Aortic root parameters were compared to measurements obtained by conventional cine acquisition, and abdominal aorta and femoral measurements were correlated between the 3D and QISS MRA acquisitions.

## Results

**Figure 1:**
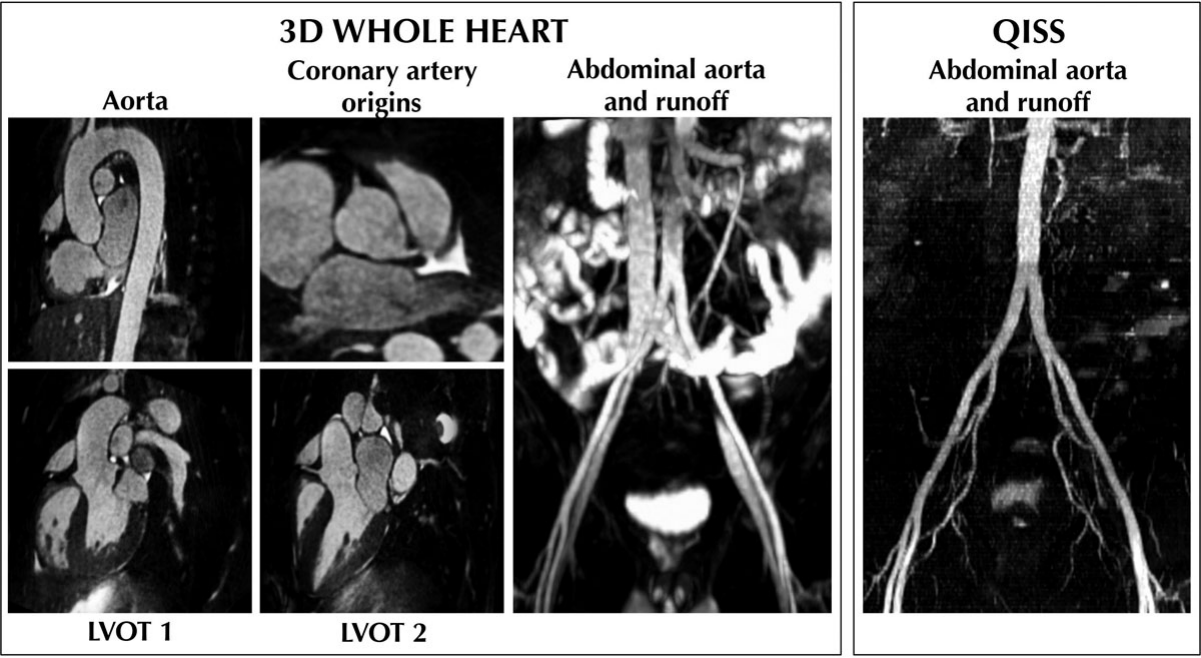


Representative thoracic and abdominal images obtained by the 3D whole heart sequence, as well as abominal MIP acquired by the QISS sequence are shown in Figure 1. The acquisition time of the thoracic 3D whole-heart scan, the abdominal 3D scan, and the abdominal QISS acquisition was 6.4±1.2, 6.3±1.1, and 3.1±0.5min, respectively. The minimum and maximum diameter, the perimeter, and the area of the aortic root were not different between the 3D whole-heart and the 2D bSSFP cine acquisitions (Table 1). No significant difference was found in the diameter of the abdominal aorta or the iliac and femoral arteries between the 3D and the QISS acquisitions (Table 1). The 3D whole heart acquisition provided a significantly better contrast-to-noise ratio (CNR) compared to the 2D bSSFP cine, however, CNR was not significantly different between images obtained by the 3D and QISS MRA protocols (Table 1).

**Table 1:** Aortic root, abdominal and femoral vascular assessment

Aortic root assessment	3D Whole Heart	2D-bSSFP	***P***
Aortic annulus diameter - min	22.3±1.7	22.6±1.5	NS

Aortic annulus diameter - max	28.2±1.4	28.1±1.4	NS

Aortic annulus perimeter	89.4±6.9	90.3±6.6	NS

Aortic annulus area	5.4±0.4	5.6±0.3	NS

CNR - valvular plane	42.3±28.4	11.9±2.7	< 0.05

CNR - aortic root	47.6±30.3	11.4±2.9	< 0.05

**Abdominal and femoral vascular assessment**	**3D Whole Heart**	**QISS**	***P***

Abdominal aorta diameter	20.8±5.5	19.9±5.7	NS

Left common iliac artery diameter	11.5±0.9	12.4±2.8	NS

Right common iliac artery diameter	11.8±1.9	10.6±4.1	NS

Left common femoral artery diameter	10.6±0.9	8.4±4.6	NS

Right common femoral artery diameter	11.2±1.1	8.8±3.6	NS

CNR - abdominal aorta	12.3±4.7	21.1±12.3	NS

CNR - left common iliac artery	13.2±8.3	20.3±9.1	NS

CNR - right common iliac artery	18.9±11.0	14.2±4.4	NS

CNR - left common femoral artery	15.2±6.9	17.9±9.2	NS

CNR - right common femoral artery	18.7±11.0	21.0±11.4	NS

CNR, contrast-to-noise ratio; NS, non-significant

## Conclusions

These preliminary results suggest that the 3D whole-heart acquisition technique provides rapid, free-breathing assessment of the cardiac and aortic root anatomy without the administration of contrast medium. Although the 3D whole-heart acquisition also enables the assessment of the abdominal vascular status, the QISS MRA provides significantly faster evaluation of the femoral access route, which improves patient compliance.

## Funding

N/A.

